# Is the Modified Dental Anxiety Scale (MDAS) a Single or Two Construct Measure? A Theoretical and Pragmatic Perspective

**DOI:** 10.3390/dj13020068

**Published:** 2025-01-31

**Authors:** Gerald Michael Humphris, Jonathan Timothy Newton

**Affiliations:** 1 Medical School, University of St Andrews, St Andrews KY16 9TF, UK; 2 Dental Public Health, Guy’s Hospital, University of London, London SE1 9RT, UK; tim.newton@kcl.ac.uk

**Keywords:** dental anxiety, theory, structural equation modelling, psychometrics, causal models, intensive longitudinal designs

## Abstract

**Background:** The MDAS questionnaire is one of a number of scales available to assess dental anxiety. It is widely used and translated into many world languages; however, it lacks an explicit theoretical backdrop to the content and structure of the measure. This paper draws upon original expositions of dental anxiety: how it develops, is maintained, and how this draws attention to a re-evaluation of the measure. To assist this inspection it was proposed to investigate a two latent construct formulation through a stepwise analysis using data from a representative survey of English respondents on their oral health (the Adult Dental Health Survey). **Aim:** To present a brief theoretical framework to underpin the measure and, as part of this study’s objectives, to provide some evidence to support the measure’s potential two-construct structure. **Method:** Narrative review, structural equation modelling, and testing of specific associations to indicate a two latent construct formulation. Data included the MDAS items (where items 1 and 2 comprise the anticipatory subscale, and items 3 to 5 describe the treatment-related subscale). These items were completed by the representative sample of respondents from the most recent Adult Dental Health Survey conducted in 2009. **Results:** The two latent construct solution for describing dental anxiety was supported. The anticipated and treatment-related subscales could be discriminated, although they were strongly correlated, demonstrating overlap. Comparison of how each construct varied across the three age groups suggests an interesting heterogeneity. In addition, the two constructs behaved differently when acknowledging previous experience of respondents’ last dental visit. Anticipatory dental anxiety was more strongly related to oral health-related quality of life (OHRQoL) than treatment-related dental anxiety as predicted. This partial evidence from empirical data and previous reports in other studies suggests that the separation of the MDAS measure into the two subscales may be warranted. **Discussion:** Researchers are recommended to report not only the total score of the MDAS in their studies but also consider presenting the two subscale scores, namely, anticipatory and treatment-related dental anxiety. Further work is indicated to determine if clinicians may find the subscales of use when assessing their patients.

## 1. Introduction

Dental anxiety remains a key psychological barrier for many people who are considering a visit to receive dental care from a dentist [1]. Early reports have noted the experience of patients when seeking dental treatment intervention [2]. It was Ruth Freeman and colleagues who published an important report that confirmed this relationship between dental anxiety and attendance in a representative sample from the UK, as presented in the Adult Dental Health Survey of 2009 [3]. She presented in the widely read British Dental Journal a multifactorial model of dental anxiety, referred to more recently in a historical review of dental anxiety as a ‘seminal paper’ on dental anxiety [4].

To further research in dental anxiety, there has been considerable attention on the measurement issues of this psychological construct. There are numerous ways to assess dental anxiety. They can be briefly summarised as consisting of behavioural, physiological, and self-report approaches. Observation scales have been developed to reveal explicit behaviours believed to demonstrate anxiety experienced by individuals within a dental clinical setting. These are chosen especially in children who may be unable to express in words their distress [5]. Similarly, the selection of physiological methods, such as skin conductance, heart rate, and cortisol blood concentration, for reflecting anxiety responses to dental surroundings and exposure to treatment has been investigated [6]. Both the behavioural and physiological methods tend to be expensive in resources, and interpretation can be challenging. Hence, for ease of administration, self-reported methods have been utilised frequently. These have included pictorial forced-choice procedures [7] and numerous rating scales collated into multi-item scales. Of the major scales that have been developed, three compilations continue to be frequently used. These include the Dental Fear Schedule [8], the IDAF-4C [9], and the MDAS [10].

The Modified Dental Anxiety Scale (MDAS) consists of 5 items in a brief questionnaire with a five-category Likert scale with an identical answering scheme across each of the self-ratings [10]. The scoring requires summing all items to furnish a total scale score ranging from 5–25. A report of the normative values of the MDAS has been prepared from a representative UK survey across 3 age bands and sex categorisation. A percentile calculator is available online for researchers and clinicians to assess the rarity of the dental anxiety value across age and sex [11]. A website lists, currently, 30 language translations that are downloadable: https://dentalanxiety.wp.st-andrews.ac.uk/ (accessed on 5 January 2025). The measure exhibits high levels of internal consistency and, where test-retest checks are conducted, provides favourable results [12]. On initial inspection, the validity of the measure would appear to support that the total scale score is assessing dental anxiety [10,12,13]. The scale has its own brief written instructions for administration, which assist consistent responsiveness to the items. It is recommended that these instructions should not be changed to retain some objectivity in the responses provided when administering the scale. Scoring to ascertain a total score is a simple summation of items.

The advantages of selecting the MDAS for both clinical and research assessment of dental anxiety can be outlined:

First, the assessment is relatively short. Some single-item questions have been devised and researched for epidemiological use [14,15]. However, the psychometrics and validity of single-item scales present a challenge, especially when making decisions on individual cases. The MDAS was derived from the 4-item original questionnaire reported by Norman Corah from Buffalo Dental Hospital. The MDAS was a modification of Corah’s scale [16] (hence the MDAS name was applied). Modifications included the addition of an item focussing on the local anaesthetic injection, a consistent answering scheme, and simplified item wording.

Second, the scale is easily administered and typically self-completed without any intervention of an assistant to coach the respondent. Furthermore, respondents rarely leave items unanswered, thereby keeping missing values to a minimum.

Third, the scale demonstrates reasonable psychometric properties for a short scale [10]. The original report of the MDAS indicated a Cronbach’s alpha coefficient of 0.91. This was corroborated by the UK representative sample [11]. Evidence for the validity of the scale is favourable, as mentioned previously. Additional research is required, however, to strengthen this evidence.

Fourth, the scale possesses a validated cut-off point to describe extreme dental anxiety [17], using a data set derived from a single university student convenience sample (n = 1108). The cut-off of 19 resulted in a sensitivity of 88% and specificity of 89%. The area under the curve from a ROC analysis was high (0.92, *p* < 0.0001). Hence, as already considered by Armfield, the Dental Anxiety Scale developed by Norman Corah was not developed to identify dental phobics *per se*, and this can be levelled at the MDAS, to some extent [18]. The MDAS classifies highly dentally anxious respondents that may, in addition, include some dentally phobics who could be selected through careful further assessment to confirm their clinical status. For example, respondents may not be suffering a high overall level of dental anxiety but simply be extremely anxious about needles. Hence, a patient may rate themselves as only moderately anxious on all items, with the exception of the item about the dental local anaesthetic, delivered via a needle injection in dental practise. The cut-off of 19 units on the total score may not identify the individual for special attention by the clinician, as they would score 5 on the local anaesthetic item and, say, ratings of 3 on the other 4 items. A summated total of 17 would not highlight the individual requiring further assessment, that is, treated as someone who can tolerate dental treatment with general support from the clinical team. Facco and Zanette raise a similar point that a cut-off is only advisory, and a patient scoring 18 on the MDAS still requires careful assessment of the suitability of dental treatment [4].

A further issue to be highlighted for users of the MDAS is the lack of a theoretical framework that has been applied to the measure. Armfield has criticised the MDAS for not expounding a theoretical background [18]. For many practical purposes, this may not be too great a problem. The dentist in a busy practice may only be interested in patients with scores approaching 19 and above on the scale measure. Such a focus may help the clinician to speed up the process of assisting patients through their dental interventions. However, for researchers who are interested in identifying factors that may be associated with dental anxiety, it is important to clarify the basis of the measurement scores. Part of this description of the theoretical basis of the MDAS is contained in the validation of the measure. This demonstrates only an implicit explanation of the measure as opposed to an explicit formulation.

Hence the aim of this paper is to present a brief theoretical framework to underpin the measure. The objectives are:

1. Provide a theoretical background to the MDAS;
2. Perform new analyses using an extant dataset to support the predicted structure and relationship to additional key variables.

Theoretical background to the MDAS measure

Reading the 5 items of the MDAS (https://dentalanxiety.wp.st-andrews.ac.uk/, accessed on 5 January 2025) will reveal that they contain questions about a future imminent visit to the dentist (items 1 and 2) and treatment procedures that require various degrees of invasiveness to the patient in the dentist’s chair (items 3, 4, and 5). The two sets of items correspond to two major elements important in identifying levels of anxiety response, namely anticipatory and situational. Current theoretical explanations of dental anxiety draw upon cognitive, behavioural, and emotional elements. The two-process model proposed by Mowrer, a general theoretical approach for the development of anxiety states, stated that phobic reactions were generated through a classical conditioning procedure (pairing of the physiological experience with the threatening situation, such as the high-speed drill or local anaesthetic injection) and maintained through operant conditioning, that is, the anxiety response or anticipation of pain or negative experience promoted avoidance of the situation [19].

The two-process model adopting stimulus (S) and response (R) pathways to explicate the development of a psychological state labelled as anxiety is recognised as being overly simplistic [20,21]. The development of dental anxiety is complex, especially as patients present to dental services with what may be viewed as appropriate levels of an apprehensive state but can shift into levels considered unusually high and described by mental health specialists as disordered. A theory of dental anxiety would need to cover both, for want of better words, normal and abnormal levels of affect. The impressive essay on mental disorder and causal explanation in psychology (and psychiatry) employing philosophical perspectives offered by Bolton, Hill & Hill [22] provides an array of explanations of how the outside world experienced by people impinges on their behaviour. The concept of ‘intentionality’ is strongly argued to be essential in understanding how individuals make sense of threatening stimuli, which a dental visit procedure would possibly engender. The representation of the threat assists the development of mechanisms to minimise, deny, or avoid thinking to reduce its exposure. Intentionality, then, can be considered similar to mental representation that causes action of an outcome. In the majority of occasions within a dental surgery context, the range of experiences and beliefs held by patients and emotional/behavioural responses are drawn into a network of representations that exerts intentionality with respect to a dental visit. Where these phenomena are moderated, it is likely that dental care can be provided without undue specialist intervention, such as anaesthesia. Where significant processes that lie outside normative existence are due to more elaborate or extreme attempts to control or cope with threat appraisals, then the receipt of dental treatment can be massively hindered. These ‘significant processes’ residing within the individual who struggles with an over-inflated sense of threat can fuel a rare but disordered response typically referred to as a phobic reaction.

The MDAS items 1 and 2 describe the individual placing themselves in the position of readiness to enter the dental surgery. The psychological effect generated through the intentionality (mental representation) of the visit can be exacerbated by the uncontrollability of the images, media reflections of traumatic scenarios, etc. The neuroscience of pain field indicates that patients anticipating a dental appointment show similar responses to actual pain prior to a visit, confirming the dental situation as highly salient [23]. The situation described in the MDAS items 3–5, namely filling, prophylaxis, and local anaesthetic, are a sample of specific stimuli that may arouse substantial threat, whether previously experienced (as traumatic) or imagined (if the person is naïve of a dental visit and various different procedures). It can be argued that the individual has more direct reference to these stimuli through the vividness and intensiveness of dental surgery images, which are often supported through social and cultural narratives [21]. The dental surgery situation is a clinical setting, very often with limited distractors in room decoration, and what images are available to the patient frequently are regarded socially as vivid (e.g., stainless steel dishes, brilliant reflective instruments, and highly radiant lighting) [24]. These representations can be difficult for individuals to control and may employ mechanisms to avoid their presence. Hence, the person with potential dental anxiety will oscillate between exerting mental effort to divert attention away from these images or find themselves in direct opposition to denial processes and focus with heightened attention. Hence, rehearsing ways of how they might manage the potential discomfort (pain or imagined tissue damage, embarrassment, or shame) of having to experience such future events. It is the dual psychological activity of ignoring or focused attending that creates the complex internal world to provide meaning (intentionality) between reality and future experience.

Patients who are dentally anxious take many visits to disconfirm the expectation of sudden or intense pain, assuming they do not avoid visiting altogether [25]. Both of the constructs defined by the items of the two subscales of the MDAS will inevitably overlap to some extent, as both are indicated by items that will promote not only the interrogations that are sparked by the item wording but also the internal-world processes triggered in both constructs. To answer items from both constructs would require, at least to some extent, accessing memories of previous experiences, for example.

The anticipatory element of dental anxiety has been linked strongly to cognitive components of uncontrollability, dangerousness, poor relationship with dental staff [26], and embarrassment [27]. Furthermore, the processes of overgeneralisation of a previous surgical negative experience and catastrophising [28] promote intentionality (in this instance: ‘exaggerated negative mental set’) and maintain dental anxiety in future visits.

In many respects the past experience of attending the dentist can be informative [29]. Patients who have been subjected to painful procedures will evoke psychological mechanisms to attempt to mitigate the affect promoted by past and recent memories. Hence, specific, anxiety-provoking mental traces are generated and encoded, which are not simply memories but representations whose intentionality predicts fear of future visits, especially at greater proximity to the clinical event (from the MDAS perspective, namely: ‘in the next day’ or ‘sitting in the waiting room’). Attempts to control, and the ability of the individual to succeed in reducing the affective quality of these representations, are believed to be involved, at least in part, in the development of dental anxiety and therefore feed into the appraisals of respondents completing ratings of the MDAS items.

The proposed theoretical formulation shares similarities with other dental anxiety models, especially the cognitive-emotional approaches [3,26,30]. The advantage of our theoretical explanation is that it specifically widens the scope to include all experiences, thoughts, images, and beliefs—real or imagined—into the framework and explicitly references the anticipatory and situational elements of the dental visit. In addition, the properties of the scale, that is, its internal consistency of the subscales, brevity, and ease of administration, enable frequent use over a timeline to test out the dynamic nature of the endogenous elements of the proposed two constructs of dental anxiety.

The MDAS questions invite a selection on a simple rating scale to assess the anxiety level of the individual respondent. The anxiety rating is graded in a progressive format using verbal descriptors. In the description of the psychometric qualities of the scale, very little has been expressed about the specific elements of the item ‘pool’. It has been argued that an advantage of the MDAS is its brevity and ease of use in surveys or the clinic. In addition, the application of classical psychometric procedures has supported a unidimensional measure with traditional reliability estimates, such as the Cronbach Alpha coefficient, as being acceptable and favourable to other existent measures. A small number of items has the benefit of not placing too great a burden on respondents but presents significant challenges to psychometricians. To break apart a relatively small battery of questionnaire items, such as the MDAS, into smaller ‘packets’ can introduce distortions in the data corpus that load less on the underlying constructs and correlate more on biases, such as systematic error, due to esoteric wording, miscomprehension, and inapplicability of general experience from respondents. Even with these cautionary issues, however, we argue that with a sufficiently sizeable data set, some of these biases can be modelled and help reveal within the MDAS the underlying two-dimensional nature of dental anxiety.

The collection of MDAS scale data has progressed since its first presentation in the research literature. As previously mentioned, there are numerous large survey studies and experimental intervention trials of innovative interventions to assist patients by reducing their dental anxiety. Furthermore, various translations in many of the major global languages have enabled a closer examination of the structure of the brief measure. Such diversity of countries and cultures from which respondents have been drawn has afforded a critical opportunity to examine empirically the unidimensional nature of the measure and whether this notion of a single ‘latent’ construct is too restrictive.

Hence, moving forward, the aim of the data-led section of this paper is to test for the presence of two distinguishable constructs within the MDAS measure. The research objectives (ROs) were:

RO1. Establish that the MDAS items can be configured as a single overall assessment of dental anxiety, thus affirming the conventional approach reported by the majority of previous studies;

RO2. Testing for evidence of two dental anxiety constructs described by the two subscales: anticipatory and treatment-related;

RO3. Support for the validity of the two construct formulation through established associations predicted by the proposed theory.

## 2. Materials and Methods

The 2009 Adult Dental Health Survey (ADHS) involved interviews and clinical examinations for a nationally representative sample of adults in England, Wales, and Northern Ireland, conducted at participants’ homes [31]. Only individuals with at least one natural tooth were eligible for the clinical examination. A team of 75 dental examiners, mainly NHS dentists, collaborated with interviewers who recorded clinical data directly into laptops. Fieldwork was split into two ten-week periods, running from October to December 2009 and from January to April 2010.

### 2.1. Sampling and Survey Design

A two-stage cluster sampling approach was used. In total, 268 primary sampling units (PSUs) were selected: 253 across England and Wales and 15 in Northern Ireland. Each PSU comprised two paired postcode sectors to minimise clustering effects. A sample of 13,400 addresses was drawn, aiming for representativeness at both national and Strategic Health Authority (SHA) levels. Data collection was split between fieldwork organisations: the Office of National Statistics (ONS) and the National Centre for Social Research covered England and Wales, while the Northern Ireland Statistics Research Agency (NISRA) handled Northern Ireland.

### 2.2. Questionnaire and Examination Content

The questionnaire and clinical examination were developed through consultation and review by consortium members, including NHS dental commissioners and university partners. Key additions included questions on NHS/private dental service access, dental anxiety (measured by the Modified Dental Anxiety Scale, MDAS), dental practice ratings, and barriers to dental care. The MDAS was introduced by the interviewer using the standardised wording: “Many people get anxious about visiting the dentist. I would like to ask you some questions about how anxious you get, if at all, with your dental visit.” The respondent was shown a card with the five response options for each of the 5 items of the scale. The respondent was invited to select their answer for each item. Socio-demographic questions, aligned with Harmonised Primary Standards for government surveys, covered education, income, employment, and ethnicity.

The clinical examination criteria were updated to identify new oral health conditions observed since the previous survey, ensuring that both the questionnaire and examination aligned with current public health needs and NHS data requirements.

### 2.3. Statistics: Testing of RO’s

RO1. The raw numerical data of five items comprising the MDAS were tested for single construct model fit (i.e., confirmatory factor analysis) using a maximum likelihood estimator. Item 1 error variance was set to unity by convention to ‘scale’ the construct. Calculations were checked to ensure all parameters were positive definite and compiled in a low number of iterations. Essential ‘fit’ indices (Comparative Fit Index CFI and Root Mean Square Error of Approximation RMSEA) were selected to assess the veracity of the model [32], and modification indices (MI) were inspected to observe potential misspecification of raw data to the measurement model. Where a large MI is returned in the output, an inspection to respecify the model was taken, and the model rerun and interpreted.

RO2. The two-construct version of the MDAS measurement model, with no cross-loadings, was run and analysed in an identical way as described in RO1, with the exception of specifying the five items into the two sub-scales with accompanying construct covariance.

RO3. Dental anxiety has a clear pattern of diminishing with age [11], so that older people (55+ years) report the least levels compared with younger adults. Our two-construct theoretical model, however, would expect a different profile of each component across age. The anticipatory elements of dental anxiety may not vary as much as those reflecting treatment-specific issues. As has been observed, the anticipation of a threat in the clinical dental setting may be pertinent to the older patient as well as the naïve. Appointments can become more complex and involved with age (e.g., fitting of surgical implants); therefore, the anticipatory dental anxiety scale may still show raised levels over age. In comparison to specific treatment procedures assessed by the treatment-related construct in which the middle-aged and the mature attender become desensitised over time [29,33,34]. Hence, we expect dissimilar trajectories of the two constructs of dental anxiety over age. This was tested by Ordinary Least Squares linear regression with age categorised into 3 levels: 16–34, 35–54, and 55+ (years). The two dental anxiety subscales were mean standardised to allow comparison.

Further associations between additional factors (whether afraid or frightened about dental visiting, past dental experience, and oral health-related quality of life) and the two dental anxiety constructs were tested using either single-way ANOVA or Pearson product-moment correlation procedures. Alpha level (2-sided) was 0.05 throughout. All analyses conducted using STATA15 [35].

### 2.4. Ethical Review and Approval

The survey adhered to rigorous ethical standards, with oversight from an NHS Research Ethics Committee (Oxford B). Ethical approval (12 June 2009) covered all aspects of the survey, including data collection protocols, consent procedures, and participant feedback mechanisms. The ADHS consortium followed the Code of Practice for Official Statistics and Government Social Research guidelines, ensuring data integrity, confidentiality, and minimising participant burden.

## 3. Results

Three sections follow that conform to each of the research objectives (ROs).

RO1. Confirmatory factor analysis demonstrated some evidence for a single construct.

The confirmatory factor analysis of the full five-item MDAS was performed using the ADHS data set (age bands: 22.3% 16–34 yrs, 35.9% 35–54 yrs and 55+ yrs 41.8%; female = 55.3%) found that a single construct was viable, taking four iterations of a maximum likelihood estimator to provide a solution. All items were related strongly to the latent construct, as shown by the item loadings ranging from 0.69 to 0.95. The LR test reflected that the observed data approximated the specified structural model with an overall fit that was considered to be fair only (chi-square = 3289.4, *p* < 0.00001). Additional conventional fit indices were consulted, and the CFI of 0.928 and the RMSEA returning a value of 0.244 were observed. These values are outside what are usually considered acceptable. The Cronbach alpha for this 5-item scale was 0.915.

RO2. On constructing a two-factor model the items gave a stronger ‘fit’.

**Figure 1:**
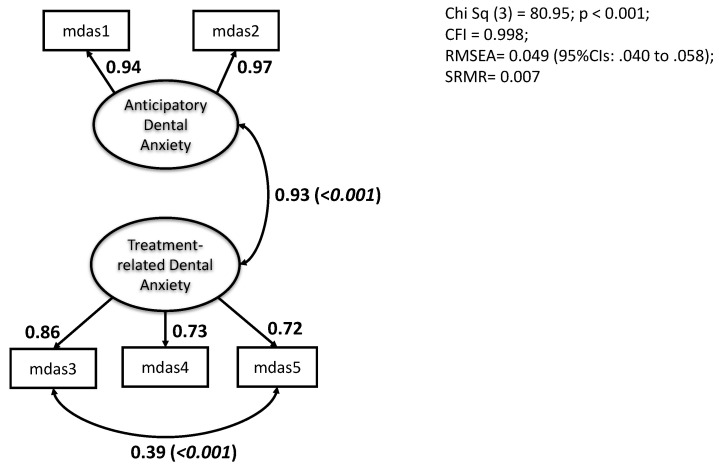
Confirmatory factor analysis of the two latent variable (ovoids) structure of the five MDAS items (rectangles). Latent variables defined by item ‘indicators’. Standardised loadings presented. Double-headed arrows show correlations (with *p* levels in brackets). Error terms excluded for clarity.

A repeat analysis was conducted and tested the two-factor theoretical model. That is, the anticipatory factor consisting of the first two items (mdas1 and mdas2) and the treatment-related factor comprising the final 3 items (mdas3 to mdas5). This solution returned rapidly after 3 iterations. The LR test gave a reduced chi-square value of 642.0, *p* < 0.00001. This value may be considered an improvement but still relatively high. The CFI and RMSEA values were 0.980 and 0.120, respectively, demonstrating that the model was improved but reflecting what some structural equation modellers regard as some ‘strain’. Modification indices were inspected, and the correlated error between the item pair mdas3 and mdas5 was significantly high and merited inclusion in the measurement model. On rerun, the model was substantially improved (Figure 1). Fit indices were considered to show a good fit (CFI = 0.998, RMSEA = 0.049 (95%CIs 0.040 to 0.058), and a chi-squared value of 80.95, *p* < 0.0001).

Compiling subscales, namely anticipatory dental anxiety (items 1 and 2 summated) and treatment-related dental anxiety (items 3 to 5 summated), resulted in a pairwise Pearson r correlation of 0.793 (95% CIs of 0.786 to 0.800). Therefore, the shared variation between these two subscales (r^2^) equalled 63%.

RO3 was separated into 4 areas.

3i. The two sub-scales described different levels of the anticipatory and situational anxiety across the age categories.

**Figure 2:**
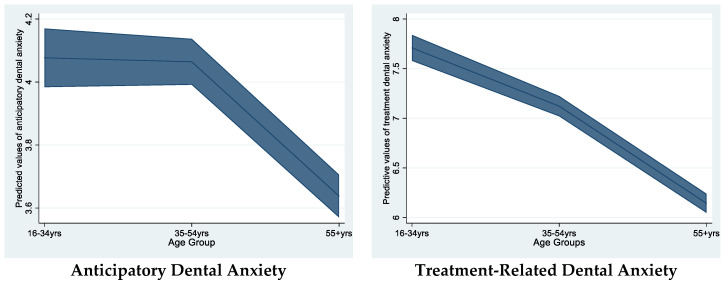
Predicted values (with shaded areas to denote the 95%CIs) of anticipatory (**left panel**) and treatment-related (**right panel**) dental anxiety, adjusted for sex across three age groups.

The relationship for age was not identical across the subscales. Anticipatory dental anxiety remained level in the low and moderate year age groups and significantly lower in the oldest age group (55+ yrs). treatment-related dental anxiety behaved differently. The levels of dental anxiety linked to treatment procedures reduced at each stage of increased age grouping (Figure 2).

3ii. The two subscales were closely associated with separate survey enquiries about being ‘afraid’ or ‘frightened’ when visiting the dentist.

It was predicted that both subscales would correlate highly with questions appearing in other areas of the survey schedule that invited comment about reasons for not attending the dentist. To the 2000 or more respondents who offered reasons for not attending the dentist due to being afraid, the variance of both subscales (anticipatory and treatment-related) explained by mentioning or not mentioning being afraid (i.e., a dichotomy) was 43% and 31%, respectively. That is, the anticipatory dental anxiety subscale was more closely associated with the voluntary admission of being afraid than the treatment-related subscale. (Table 1) Both sub-scales were divided by the number of items to enable comparison on the same metric, that is, 1 to 5.

Likewise, when the survey respondents were invited to give any other comment they wished at the end of the interview, just over 100 respondents stated being frightened of visiting the dentist out of the 1900 (approximately) who volunteered other information (e.g., could not find an NHS dentist). The 102 respondents who mentioned being frightened were significantly (*p* < 0.001) more dentally anxious on both subscales than those who mentioned other issues.

3iii. However, the levels of dental anxiety on either subscale (anticipatory or treatment-related) were sensitive to experience received at their last dental visit, including if the visit consisted of a check-up, filling, or receiving advice about looking after teeth.

This interesting set of results was detected by testing against some theoretically relevant responses from those people who answered survey questions about their last visit to the dentist. (Table 2) From our theoretical perspective, we would expect a respondent’s treatment-related dental anxiety (TRDA) not to differ according to whether they had received a check-up or not. The check-up experience would not help extinguish anxiety towards specific dental treatment procedures, whereas those who were given advice at their last dental visit might report increased treatment-related dental anxiety due to fear that they might not have followed advice and suffer consequential invasive treatment.

3iv. Oral Health-Related Quality of Life (OHRQoL) associated differentially with the two components of dental anxiety.

The individual’s rating of OHRQoL was recorded in the survey and scored so that negative impacts on the respondent would return a higher score. We expected a positive association between these impact ratings and dental anxiety. More specifically, we predicted a stronger association with apprehension of future visits rather than separate procedures of dental treatment intervention. The Pearson correlations between each of the subscales, anticipatory and treatment-related, with OHRQoL were 0.204 and 0.169, respectively. The correlations were not identical (z = 2.69, *p* < 0.007) and went with prediction.

## 4. Discussion

The main aim of this paper has been to present a theoretical exposition for a two-construct formulation for the assessment of dental anxiety using the MDAS measure. A narrative theoretical underpinning of the measure has been presented. A UK data set has been utilised to examine some of the predicted associations arising from this new framework. Future strategies for research are considered to promote our understanding of dental anxiety.

### 4.1. MDAS Assessment: One or Two Constructs?

The unidimensionality of the MDAS as a measure of dental anxiety can be confirmed from the research that has administered the scale. Hence, previous reports presenting total scale results are important milestones in assisting our general knowledge of dental anxiety. The items comprising the measure, however, on detailed inspection, divide clearly into two sets of questions. They have been labelled as anticipatory and treatment-related. Opportunity to test this formulation was provided by the latest Adult Dental Health Survey. The survey adopted the MDAS measure of dental anxiety, enabling an ample representative sample to test the two-construct structure. As predicted from the close item inspection, the proposed two constructs were identified and possessed sufficient internal consistency to be reported as separate sub-scales. The confirmatory factor analysis results were reassuring by showing strong evidence of two constructs. Two points of caution need to be expressed. First, a correlated error between items 3 and 5 of the MDAS needed to be entered into the structural model to return good fit indices (CFI > 0.98; RMSEA < 0.05; SRMR < 0.05). This was considered in our introduction as a likely scenario, especially when a small number of items constitute a hypothesised construct. In the first paper to present UK norms for the MDAS measure, the confirmatory factor analysis required three correlated errors for a ‘close’ fit of the 5-item unidimensional scale. It is interesting that on reflection this demonstrated a degree of ‘strain’ in the measure [36]. With this ADHS data set we have shown that a good fit can be secured with separating the measure into 2 constructs with the inclusion of a single correlated error. The second note of caution to raise is the high degree of overlap in the two subscales, as shown statistically by the 63% of shared variation. We argue for a compromise, therefore, in handling MDAS statistical presentation. For pragmatic purposes, the total MDAS score should be reported to enable comparison to previous research and also to provide an indicator of respondents who score especially high (greater than or equal to 19 out of the maximum of 25). In addition, the subscale statistics can be tabulated to supply additional information on two potentially important and theoretically defined features of dental anxiety, namely anticipatory and treatment-related.

### 4.2. Issues of Validity of the Two-Construct MDAS Measure

The confirmatory factor analytical solution for the MDAS single and two-construct models of dental anxiety demonstrated a satisfactory fit for the former and a good, if not improved, fit for the later model as previously mentioned. Support for a two-construct dental anxiety structure within the MDAS was found by exploring the relationship of each construct with other variables. First, the different pattern of how the two constructs related to age was considered an important opportunity to test if the subscales related to age identically. It is understood that general anxiety reduces with age in adulthood. Some evidence suggests that trait anxiety is less resistant to change, but some reduction in much older adults (55+ years) may be expected [37]. Situational anxiety, however, may be more receptive to change, as over the life span the real or imagined experience of feared objects is greater, and therefore, through the instances of each contact, the associated anxiety diminishes through a process of dissemination [38]. A report that feared images lose psychological strength in affect in older people is also consistent with this explanation of reductions in reported situational anxiety [37,39]. The evidence of anxiety diminishing over the age range, however, is very complex, as Teachman *et al*. have shown in their longitudinal study, based in Baltimore, of anxiety changes in young and older adults. When physical threat is considered, individuals of older age tend not to show such a reduced level of situational anxiety [40]. Hence, researchers are alerted to the possibility that not all patients attending the dentist, even if older, will demonstrate a sanguine assessment of what they might conceive would be a threatening intervention.

As predicted, there was a significant (*p* < 0.001) raised dental anxiety observed for each of the two MDAS sub-scales, with approximately 440 respondents affirming that they were afraid of the dentist as a reason for not visiting the dentist (Table 1) compared with those (n = 1600+) who gave other reasons (e.g., cost of treatment). The strength of the association was not identical, however, for each of the two subscales. This observed disparity supports the view that the subscales measure a somewhat different construct.

Those who stated that they had a check-up at their last visit are likely to be more regular attendees and are less anxious about visiting the dentist, the general experience of meeting staff, the setting, and possible treatment. We did not expect a check-up to influence treatment-related dental anxiety, as a simple inspection of the mouth would not present the threatening experience to act as stimuli to extinguish anxiety to specific dental procedures.

From a broad theoretical stance, it could be predicted that when a respondent at their last visit experienced a filling, they might respond with an increased level of treatment-related dental anxiety. This was not observed, however. Two possible explanations can be offered, the first functional and the second, theoretical. First, the experience of sustaining a filling at the last visit perhaps was less traumatic than expected, and therefore a relative lowering of treatment-related dental anxiety was found, i.e., a desensitising experience. From our two-construct theoretical perspective, the second explanation, the individual actively denies the negative affect resulting from the filling procedure (due to avoidance), but anxiety generalises to the broader construct of anticipatory dental anxiety. Such phenomena of stimulus generalisation are widely recognised phenomena in the individual’s repertoire of attempts to manage a traumatic experience, regardless of setting.

Advice giving at the last dental visit raised levels of dental anxiety in comparison to those not receiving advice. Respondents provided with advice may have been told that if advice was not heeded, then future dental problems may result, requiring invasive treatment. In addition, accepting advice from a dentist demands a certain level of trust in the health professional by the patient. As trust is closely linked to dental anxiety [41,42], a lack of trust might impact more strongly on their estimate of dental treatment being necessary. This conjecture would be consistent with the raised treatment-related dental anxiety subscale score in those reporting being given advice compared to those who were not.

To place into context the findings of Table 2, it will be apparent that post hoc formulations were utilised. Hence, caution is required, and further investigation with appropriately powered samples will confirm the presented effects that admittedly were fairly small. We argue that some basic testing of associations between patient experiences and subsequent anxiety ratings may reveal helpful confirmation of our approach of theorising a two-construct formulation. As mentioned later, the studies that may best assist theory development are the intensive longitudinal approach [43] with key experiences embedded during the timeline of the frequent anticipatory and treatment-related dental anxiety assessments.

The link between dental anxiety and OHRQoL was confirmed in our analyses. This result corresponds with previous reports of patients with high dental anxiety who have corresponding low OHRQoL [44]. Those patients who anticipate threat on any proposed visit, categorised as the generally dentally anxious patient [45] will commonly avoid going, and their OHRQoL will deteriorate.

### 4.3. Previous Work Using the Two-Construct Version of the MDAS

The MDAS subscales were associated differentially with two aspects of general anxiety (using the Hospital Anxiety and Depression Scale-Chinese translation) in a sample of dental attenders at a Beijing dental hospital. Specifically, the construct defining negative affectivity (that is, the degree to which the individual’s emotional perspective of most experiences tends to be reflected adversely as opposed to positively) [46] correlated strongly on the anticipatory two-item subscale and poorly on the treatment-related three-item subscale. The model presented with the Chinese data specifically purports that the anticipatory dental anxiety construct ‘causes’ the treatment-related dental anxiety construct. Our model, crucially, does not specify one construct ‘causing’ another. In fact, we argue that a bi-directional relationship exists between the two constructs. So that either construct can ‘cause’ the other. In the structural equation modelling field, this is known as a non-recursive model and the special case of a ‘reciprocal causative process’ [36]. Such models require at least two waves (and preferably more) to ‘identify’ (that is, produce a stable solution) and return fixed estimates compiled through a process of iterative calculations.

Lahti and colleagues at her laboratory at the University of Turku and elsewhere in Finland have investigated dental anxiety using the MDAS. An early study drawing on the Finn Brain database has shown convincingly that general anxiety (Symptom-90 anxiety subscale) was more strongly associated with anticipatory than treatment-related dental anxiety. The study was based on a substantial data corpus of thousands of adult respondents and sophisticated data analytical techniques [38]. Two further reports from Lahti’s group of a correlational nature are especially interesting and strongly support the MDAS two-construct model. The first is a survey that focused on alexithymia, which is considered the inability to experience, verbalise, or name emotions. The analysis confirmed for both women and men that there were different associations of the two constructs of dental anxiety with alexithymia [39]. The second study, utilising data from 428 participants, found that sensory sensitivity and pain catastrophising were independently associated with anticipatory and treatment-related dental anxiety, while difficulty identifying feelings was not [47].

Apart from studies that provide support for the two-construct formulation of the MDAS through inspection of relevant associations, there is also the specific case of intervention effects to reduce dental anxiety. Separation of the MDAS into two subscales has appeared to assist interpretation of a novel intervention. In a recent RCT study, Lahti and colleagues tested the effect of a brief virtual reality experience prior to a dental visit requiring some treatment. They analysed the MDAS data (Finnish translation) into the same two dental anxiety constructs. The influence was detected by the anticipatory subscale but not the treatment-related subscale [48].

The proposal that the two constructs are likely to reciprocate their influence on each other is predicted by the theoretical approach highlighted in the background section. This relates to our intentionality proposal of how dental anxiety develops and is maintained [22]. Of course, any theory needs to explicate not only how it might develop but also reveal the processes of maintenance. To some extent this is met in the model of dental anxiety maintenance outlined by Armfield [49]. His ‘dental anxiety—attendance circuit’ sets out a convincing scenario of dental anxiety being triggered by some stimulus (for example, pain or embarrassment on visiting the dentist) that triggers avoidance and hence maintains, if not increases, dental anxiety. Hence, development is dependent on internal and exogenous factors. There is also, we believe, some merit in considering that a specific different interior and parallel process might occur where separate elements of dental anxiety ‘feed’ or alternatively ‘inhibit’ each other. This endogenous or internal set of states may already be predicted even though it is acknowledged that the processes are poorly understood and less investigated. With the ability to assess separate elements of dental anxiety, as illustrated here with the MDAS two-construct structure, it is possible to take estimates frequently over a time duration to study deliberately the causative or reciprocal interrelatedness of these constructs and posit tentatively a causative pathway.

### 4.4. Limitations

The small number of items comprising the two subscales of the MDAS should encourage researchers to seek additional evidence to support the two-construct measurement model. In addition, we have used only a single data set, albeit extensive and representative, to empirically test some aspects of the underlying validity of the two-construct measurement model.

### 4.5. Future Research Strategy to Assist Understanding of the Dynamics of Dental Anxiety Development over Time

An important research theme for future investigation is to collect multiple ratings of dental anxiety (using the MDAS, for instance) and plot changes over significant passages of time coincident with dental attendance experience (such as the introduction of a new dental service) or other such external experiences such as a public education campaign [50]. The resultant data sets can then begin to profile the two constructs and to introduce additional ratings for investigation, such as pain experienced during the course of the duration of time being investigated. For example, a study to reflect whether patients embarking on the intrusive surgical procedure of implant placement might be investigated in detail—by weekly ratings completed via mobile phone—to understand the factors that produce not only the final outcome of the dental anxiety estimate at study end but also the interaction between the two constructs of dental anxiety outlined and the influence, if any, on pain experienced over the course of the multiple surgical visits and immediate follow-ups following surgical intervention. The introduction of longitudinal intensive analytical methods [43] is able to unlock the shared variation and explore causative models that are testable with these statistical approaches (e.g., cross-lagged latent variable series) that are becoming more commonplace [51]. The scenarios that can be investigated enable what Wide and Wide-Boman describe, using dental anxiety as a case example, as a ‘causal analytical investigation’ [52]. The selection of the interval between each rating (daily, weekly, or monthly) will depend, crucially, on the likely theoretical changes in dental anxiety that might be expected to respond following a key event (e.g., implant surgery) or the introduction of a new dental service or system of care. We would suggest that when investigating a new dental service, changes in dental anxiety across time can be collected practically through a monthly enquiry. Such a research design enables pragmatic causation to be explored.

In conclusion, our intentionality theory of dental anxiety to underpin the MDAS is proposed. It is inclusive of the whole range of dental anxiety reporting, from mild to moderate, to include extreme levels, containing those with severe complaints, and in rare cases, mental disorders. Two different aspects of dental anxiety can be reliably captured, each with some evidence to support validity.

## Figures and Tables

**Table 1:** Means of each dental anxiety subscale (divided by number of items) according to whether the respondent mentioned that their reason for not attending the dentist was due to being ‘afraid of going’.

	Anticipatory Dental Anxiety						Treatment-Related Dental Anxiety					
	Not Mentioned		Mentioned				Not Mentioned		Mentioned			
	Mean	SD	Mean	SD	F	*p*	Mean	SD	Mean	SD	F	*p*
**Reason for not going to the Dentist?**												
**Afraid of going**	1.89	1.14	4.25	0.98	1606	<0.001	2.22	1.15	4.02	0.92	917.8	<0.001
**N**	1733		441				1637		439			

**Table 2:** Means of each dental anxiety subscale presented across 3 dental experiences at respondents’ last dental visit.

	Anticipatory Dental Anxiety						Treatment-Related Dental Anxiety					
	Yes		No				Yes		No			
	Mean	SD	Mean	SD	F	*p*	Mean	SD	Mean	SD	F	*p*
**At last visit**												
**Had check up**	1.92	1.18	2.08	1.34	22.54	<0.001	2.28	1.14	2.31	1.23	1.33	0.25
**N**	9698		1450				9578		1393			
**Had teeth filled**	2.08	1.27	1.89	1.18	50.6	<0.001	2.31	1.17	2.27	1.14	2.05	0.152
**N**	2982		8160				2965		7999			
**Had advice about looking after teeth**	1.96	1.2	1.92	1.21	2.76	0.1	2.35	1.15	2.23	1.15	32.33	<0.001
**N**	4913		6193				4848		6084			

## Data Availability

This paper used a public database available on request. Details can be found on http://doi.org/10.5255/UKDA-SN-6884-2, (accessed on 5 January 2025).
